# Pulmonary cystic disease in HIV positive individuals in the Democratic Republic of Congo: three case reports

**DOI:** 10.1186/1752-1947-1-101

**Published:** 2007-09-22

**Authors:** Steven FJ Callens, Faustin Kitetele, Patricia Lelo, Nicole Shabani, Jean Lusiama, Okitolanda Wemakoy, Robert Colebunders, Frieda Behets, Annelies Van Rie

**Affiliations:** 1 School of Public Health, University of North Carolina at Chapel Hill, USA; 2 Pediatric Hospital Kalembe Lembe, Lingwala, Kinshasa, Democratic Republic of Congo; 3 School of Public Health, University of Kinshasa, Democratic Republic of Congo; 4 University of Antwerp, Belgium and Institute of Tropical Medicine, Antwerp, Belgium

## Abstract

Pulmonary emphysema and bronchiectasis in HIV seropositive patients has been described in the presence of injection drug use, malnutrition, repeated opportunistic infections, such as *Pneumocytis jirovici *pneumonia and *Mycobacterium tuberculosis *infection, and has been linked to the presence of HIV virus in lung tissue. Given the high burden of pulmonary infections and malnutrition among people living with HIV in resource poor settings, these individuals may be at increased risk of developing pulmonary emphysema, potentially reducing the long term benefit of antiretroviral therapy (ART) if initiated late in the course of HIV infection.

In this report, we describe three HIV-infected individuals (one woman and two children) presenting with extensive pulmonary cystic disease.

## Background

A number of reports from the US and Europe have described cystic lesions in the lungs of children and adults infected with the human immunodeficiency virus (HIV) with or without overt acquired immunodeficiency syndrome (AIDS) [1,2]. Pulmonary emphysema, an anatomic alteration of the lung, is characterized by abnormal enlargement of the air spaces distal to the terminal bronchiole, with destruction of their walls and without obvious fibrosis. In industrialized countries, a 15 to 42% prevalence of HIV related pulmonary emphysema has been observed in small case series, much higher than the estimated 1.8% prevalence in the general population [1]. Bronchiectasis is defined as irreversible dilatation of the bronchi, usually associated with inflammation. In a cohort of 164 HIV infected children from New York, the prevalence of bronchiectasis was estimated at 15% [2].

Little is known about the pulmonary emphysema and bronchiectasis in people living with HIV from resource poor settings. We report on three HIV seropositive patients (one adult and two children) with pulmonary emphysema seen at the pediatric Hospital Kalembe Lembe in Kinshasa, Democratic Republic of Congo and review the literature on HIV-associated pulmonary emphysema.

## Case presentation

### Case 1

A 39-year old HIV seropositive woman, with a medical history of recurrent oral ulcerations, thoracic *Herpes zoster*, weight loss, caries, repeated pneumonia and three episodes of smear positive tuberculosis (1990, 2001 and 2003), presented in our clinic in May 2005 with chronic productive cough and intermittent hemoptysis. Her family history did not reveal any hereditary lung diseases. She had never smoked or used intravenous drugs. She received cotrimoxazole prophylaxis but had never had access to ART.

Clinical exam revealed a normal respiratory and cardiac rate, weight 47.3 kg and height 159 cm (body mass index; BMI: 18.7), pallor, lesions of an old zona eruption on the left shoulder, oral ulcerations and caries. Lung auscultation revealed discrete crepitations in both lung fields. The CD4^+ ^T lymphocyte count was 329 cells/mm^3^.

**Figure 1:**
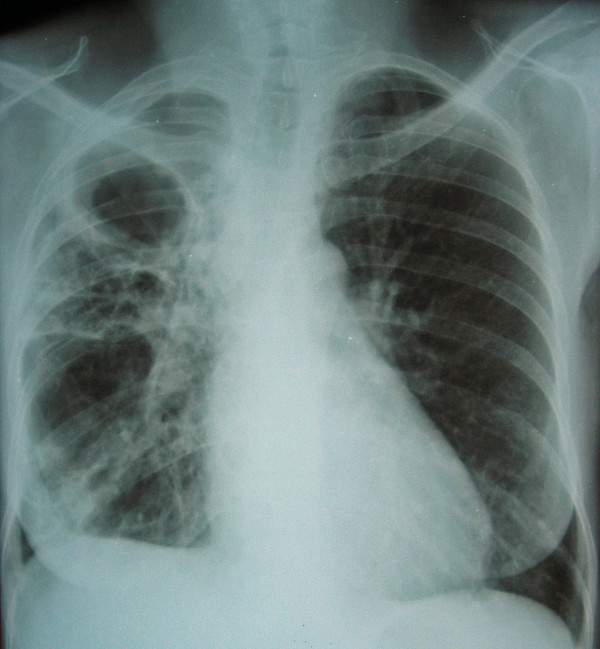
Case 1: Surinfected dystrophic bullous emphysema with pleurisy on the right side.

A sputum specimen was negative for acid fast bacilli. Gram stain showed a large number of epithelial and polynuclear cells, gram negative bacilli and gram positive cocci. Sputum culture grew *Klebsiella pneumoniae*, sensitive to nalidixic acid, norfloxacin, ciprofloxacin, cefotaxime and gentamycin. Chest X-ray (Figure 1) was compatible with surinfected dystrophic bullous emphysema and a small right-sided pleural effusion.

The patient was treated with seven days of clindamycin, followed by one week of amoxicillin/clavulanic acid, with good clinical response. ART (stavudine, lamivudine, nevirapine) was initiated, based on WHO stage III clinical disease. Because hemoptysis persisted and pulmonary infiltrates increased, treatment with fluconazole for presumptive fungal surinfection was started 2 weeks after initiation of ART with resolution of symptoms and signs.

### Case 2

A 4 year old HIV seropositive girl, with a history of oral ulcerations, presented at our clinic in April 2005. She was on cotrimoxazole prophylaxis, but never had access to ART. On the first visit, she presented with diarrhea, cough and rhinorrhea. She was severely malnourished, with discolored hair and distended abdomen, body weight was 8 kg for 91 cm (BMI: 9.7). Clinical exam revealed normal cardiac frequency (90 beats/min), a borderline respiratory rate (40 cycles/min), bilateral ronchi, drumstick fingers and generalized lymphadenopathy.

CD4^+ ^T lymphocyte percent was 3.5% and CD4^+ ^T lymphocyte count 92 cells/mm^3^. She was initially treated oral clindamycin and subsequently with intramuscular ceftriaxone, oral salbutamol, prednisolone and quinine, with good clinical response. The child started ART (stavudine, lamivudine, nevirapine) in May 2005.

In June 2005, after bathing in a river, she developed severe dyspnea, persistent cough, vomiting and fever. Clinical exam revealed a fever (38.7°C), tachypnea (80 cycles/min) and tachycardia (170 beats/min). Lung auscultation revealed crepitations, ronchi and wheezing. She was treated for possible aspiration pneumonia with ceftriaxone and gentamycin, hydrocortisone, acetaminophen, and salbutamol.

**Figure 2:**
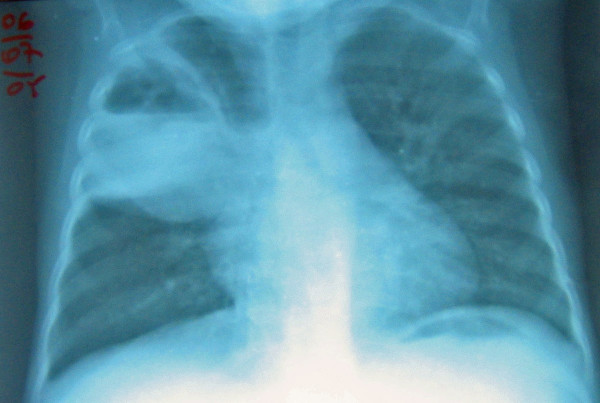
Case 2: Pyopneumothorax in an extensive bulla.

In July 2005, the child presented with cough, dyspnea and fever. There was tachypnea (56 cycles/min), tachycardia (140 beats/min) and tubar sound on auscultation. Chest X-ray (Figure 2) showed a pyopneumothorax in an extensive bulla on the upper right side and the child was started on clindamycin and gentamycin. The pyopneumothorax resolved, but the cough and fever remained. The child was started on ipratropium and budesonide inhalation, antituberculous treatment and nutritional rehabilitation. One month later, she was asymptomatic and the bulla had dramatically reduced in size.

### Case 3

A 13 year old HIV seropositive child presented in our clinic in June 2005, with a history of persistent ductus arteriosus, epistaxis, oral ulcerations, oral candidiasis, prurigo and pulmonary tuberculosis,. She was on co-trimoxazole prophylaxis but had never had access to ART. Clinical exam revealed a body weight of 19 kg for a height of 122 cm (BMI: 12.8), an umbilical hernia and prurigo.

The CD4^+ ^lymphocyte percentage was 12%, absolute CD4^+ ^T lymphocyte count was 356 cells/mm^3^. ART (stavudine, lamivudine, nevirapine) was started in July 2005.

**Figure 3:**
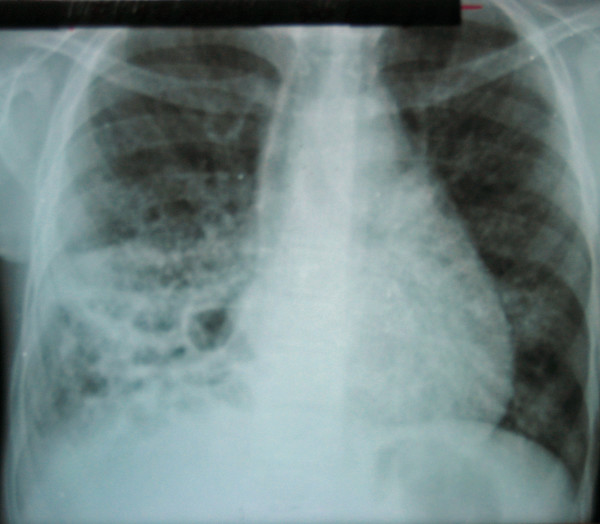
Case 3: Alveo-interstitial infiltrate in right lower filed with multiple bullae spread throughout right lung field.

In August 2005, she complained of nocturnal fever and transpiration since 7 days, epistaxis, anorexia, a productive cough and mucopurulent sputa for 2 weeks. The clinical exam revealed a normal respiratory rate (28 cycles/min), a cardiac rate of 92 beats/min, seborrheic dermatitis, cheilitis and ronchi most pronounced at the base of the right lung. A thick smear was negative; white blood cell count was 17.600 cells/mm^3 ^with 48% neutrophils and 52% lymphocytes. Sputum smear examination was negative for acid fast stain, gram stain showed epithelial cells, polynuclear cells, yeast buds, and *Candida albicans *growth in sputum culture. Amoxicillin, quinine, fluconazole and multivitamin were prescribed. Two weeks later, the child returned because of persistent cough and fever. Clinical exam showed a fever (39°C), a normal respiratory rate (25 cycles/min) and bilateral ronchi on auscultation. Chest X ray (Figure 3) showed an alveo-interstitial infiltrate in the right lower lung field and multiple bullae spread throughout the right lung.

Early September 2005, the girl died due to complicated measles infection.

## Discussion

We report three cases of pulmonary cystic disease in HIV positive individuals with multiple risk factors for the development of emphysema.

Although emphysema and bronchiectasis are defined by histopathological exam of lung biopsies, a diagnosis can be inferred based on chest X-ray, pulmonary function tests, and high resolution computed tomography (HRCT). In the absence of HRCT, as was the case in our centre, the exact diagnosis might be difficult to establish. Emphysema is only consistently diagnosed on chest X-ray when the disease is severe, diagnosed in about half the instances when the disease is of moderate severity and almost consistently missed in mild cases [3]. Moreover, bullae reflect only locally severe disease and are not necessarily indicative of widespread emphysema. Bullae on conventional chest X-ray present as radiolucent areas larger than one centimeter in diameter surrounded by arcuate hairline shadows. In bronchiectasis, findings may be nonspecific and include focal pneumonitis, scattered irregular opacities, linear or plate-like atelectasis, or specifically dilated and thickened airways that appear as ring-like shadows or tram lines [4].

Several potential risk factors, such as intravenous drug use, repeated pulmonary infections, malnutrition and HIV infection itself have been described, but no rigorous case control studies have been published.

Intravenous injection of a number of oral medications has been associated with the development of talcosis and subsequently emphysema and bulla formation. Intravenous illicit drug use, methylphenidate in particular, has been linked to the development of a specific pattern of lower-lobe emphysema, resembling panacinar emphysema due to α_1_-protease inhibitor deficiency [5].

Recurrent infections, especially *Pneumocystis jiroveci*, have been associated with emphysematous changes and bronchiectasis in patients with HIV infection [2]. Conversely, while tuberculosis is one of the most prevalent opportunistic infections in people living with HIV in resource poor settings, the association with emphysema and bronchiectasis has not been studied widely in this population [6].

Emphysema may also be a direct effect of the HIV virus. Reported by Diaz et al, four HIV infected patients without history of pneumonia, other pulmonary opportunistic infections or other known causes of emphysema, showed a markedly abnormal pulmonary function with air-trapping, hyperinflation, decreased diffusing capacity and emphysema-like bullous changes on HRCT [7]. In one autopsy study of AIDS patients, infection of macrophages by the HIV virus resulted in the upregulation of matrix metalloproteases in neighboring uninfected macrophages and was strongly associated with loss of alveolar wall [8].

Lymphoid interstitial pneumonitis (LIP) has been suggested as an etiologic factor for bronchiectasis and bullae in HIV positive patients. Two patients with transbronchial biopsy evidence of LIP but no antecedent histories of infection have been reported to have CT-proven bronchiectasis [9]. One case report described extensive bullae in the course of LIP [10].

Malnutrition and weight loss, common complications of HIV infection in resource poor settings, may affect human lung structure and function, as reported in starvation [11]. While some authors argue that some of these changes may be reversible, the chronic catabolic state in HIV infection and insufficient calorie intake in sub Saharan Africa may lead to irreversible lung damage and chronic pulmonary disease [11]. Furthermore, malnutrition increases the risk of infections in general [12].

Treatment options for emphysema and bronchiectasis are focused on symptom control, treatment of disease exacerbation and attempts to limit the rate of disease progression. In the absence of lung function tests, the assessment of clinical improvement following medical intervention will be limited and rely solely on patient history. Medical interventions include smoking cessation, bronchodilators, oxygen supplementation, and management of exacerbations with antibiotics [13]. HIV positive patients should also be offered cotrimoxazole prophylaxis, isoniazid preventive therapy, and could benefit from vaccination against *Haemophilus influenzae *and *Streptococcus pneumonia *[14]. Given the direct cytotoxic effect of HIV infection, inhibition of HIV replication at the lung compartment through ART may further modulate the disease [8]. It remains however, unclear if ART should be considered in all HIV infected patients with proven emphysema or bronchiectasis, independent of CD4 count and clinical staging.

The commonest indication for surgical bullectomy is severe dyspnea in the setting of a large bulla occupying at least 30 percent of the hemi thorax. Although, lung volume reduction surgery improves survival compared to conservative therapy, it should be reserved for patients with upper-lobe-predominant emphysema in well-equipped and experienced treatment centers [15].

## Conclusion

A high (15 to 42%) prevalence of emphysema and bronchiectasis among people living with HIV has been reported in industrialized countries, but no data is available form resource poor settings. Given the high incidence of pulmonary infections and malnutrition, HIV positive persons in resource poor settings are at an even higher risk of developing pulmonary emphysema and cystic disease. Chronic pulmonary disease, including emphysema and bronchiectasis, might become an increasingly important HIV associated co-morbidity. Due to the irreversible and progressive nature of the chronic lung disease, these pulmonary complications could compromise the benefits of expanding access to ART in resource poor settings.

## Competing interests

The author(s) declare that they have no competing interests.

## Authors' contributions

SC wrote the manuscript; PL, NS, JL provided the case reports and reviewed clinical data described in the manuscript; OW, RB, FB and AVR provided scientific input in the discussion of the article. All authors read and approved the final manuscript.
